# Transcriptomic analysis of genes related to alkaloid biosynthesis and the regulation mechanism under precursor and methyl jasmonate treatment in *Dendrobium officinale*

**DOI:** 10.3389/fpls.2022.941231

**Published:** 2022-07-22

**Authors:** Chunyan Jiao, Mengke Wei, Honghong Fan, Cheng Song, Zhanjun Wang, Yongping Cai, Qing Jin

**Affiliations:** ^1^School of Life Sciences, Anhui Agricultural University, Hefei, China; ^2^College of Life Sciences, Hefei Normal University, Hefei, China; ^3^College of Biological and Pharmaceutical Engineering, West Anhui University, Luan, China; ^4^State Key Laboratory of Utilization of Woody Oil Resource, Hunan Academy of Forestry, Changsha, China

**Keywords:** *Dendrobium officinale*, alkaloid biosynthesis, precursors, methyl jasmonate, transcriptome

## Abstract

*Dendrobium officinale* is both a traditional herbal medicine and a plant of high ornamental and medicinal value. Alkaloids, especially terpenoid indole alkaloids (TIAs), with pharmacological activities are present in the tissues of *D. officinale*. A number of genes involved in alkaloid biosynthetic pathways have been identified. However, the regulatory mechanisms underlying the precursor and methyl jasmonate (MeJA)-induced accumulation of alkaloids in *D. officinale* are poorly understood. In this study, we collected *D. officinale* protocorm-like bodies (PLBs) and treated them with TIA precursors (tryptophan and secologanin) and MeJA for 0 (T0), 4 (T4) and 24 h (T24); we also established control samples (C4 and C24). Then, we measured the total alkaloid content of the PLBs and performed transcriptome sequencing using the Illumina HiSeq 2,500 system. The total alkaloid content increased significantly after 4 h of treatment. Go and KEGG analysis suggested that genes from the TIA, isoquinoline alkaloid, tropane alkaloid and jasmonate (JA) biosynthetic pathways were significantly enriched. Weighted gene coexpression network analysis (WGCNA) uncovered brown module related to alkaloid content. Six and seven genes related to alkaloid and JA bisosynthetic pathways, respectively, might encode the key enzymes involved in alkaloid biosynthesis of *D. officinale*. Moreover, 13 transcription factors (TFs), which mostly belong to AP2/ERF, WRKY, and MYB gene families, were predicted to regulate alkaloid biosynthesis. Our data provide insight for studying the regulatory mechanism underlying TIA precursor and MeJA-induced accumulation of three types of alkaloids in *D. officinale*.

## Introduction

*Dendrobium officinale* Kimura et Migo, belonging to the Orchidaceae family, is a rare perennial herb that has antiaging and hypoglycemic properties and is very effective in the treatment of gastrointestinal diseases (Guo et al., 2020). *D. officinale* is listed separately in the Chinese Pharmacopeia (2010 edition), indicating that it is nationally recognized for its medicinal value (Tang et al., 2017). Several active ingredients, including polysaccharides and alkaloids, reportedly make major contributions to the excellent medicinal effect of *D. officinale* (Xu et al., 2013). To date, most research has focused on the characterization of polysaccharides, and few studies have examined alkaloids due to their complex chemical compositions and diversity. Some previous studies have shown that the main types of *D. officinale* alkaloids are pyridines, isoquinolines, purines, amides, and terpenoid indole alkaloids (TIAs) (Mou et al., 2021), with TIAs, such as sempervirine, glycoperine, carapanaubine, and quinine, being the most abundant (Jiao et al., 2018; Cao et al., 2019).

TIAs are a group of natural products with important biological activities. More than 4,000 such alkaloids have been derived from plants in the families Apocynaceae, Rubiaceae, Loganiaceae, and Nyssaceae (Huang et al., 2016). Although the structure of TIAs is complex, they all originate from a common intermediate, strictosidine, which is a product of the condensation of two intermediates, tryptamine and secologanin, catalyzed by the enzyme strictosidine synthase (STR) (Liu et al., 2021). The TIA content in medicinal plants is often low, and the extraction of these compounds is difficult. Moreover, the shortage of natural medicinal plant resources seriously affects the development and utilization of medicinal plants. Adding exogenous substances, such as precursors, elicitors, nutrient elements, and signal molecules, to plant tissue culture systems is an effective method to improve the plant TIA content (Jeet et al., 2020). For example, the accumulation of vinblastine was improved by treatment with tryptophan (Trp) in multiple shoot and callus cultures of *Catharanthus roseus* (Sharma et al., 2019), and MeJA and JA can be used as signal molecules to regulate the biosynthesis of plant secondary metabolites, such as nicotine, anthocyanin, artemisinin, and TIAs (Wasternack and Strnad, 2019).

In the last few years, transcriptomic technology has become a widely used tool to investigate the biosynthesis and regulatory mechanisms of secondary metabolites. The first transcriptomic analysis paper focused on *D. officinale* was published in 2013. It revealed several putative alkaloid biosynthetic genes and transcription factor (TF) genes in *D. officinale* (Guo et al., 2013). To date, a large number of genes involved in alkaloid biosynthesis in *D. officinale* have been identified by transcriptomic analysis (Shen et al., 2017; Chen et al., 2019; Wang et al., 2020, 2021); however, the regulatory mechanism underlying the accumulation of alkaloids, especially TIAs, in TIA precursor and MeJA-treated *D. officinale* is largely unknown. In this study, the *D. officinale* protocorm-like bodies (PLBs) were treated with TIA precursor and MeJA. Then, total alkaloid content of treated and non-treated PLBs was measured, and we used RNA sequencing (RNA-Seq) analysis to study variation in gene expression between the treatment group and control group. Furthermore, WGCNA was used to screen the key modules involved in alkaloid biosynthesis, and the expression of related genes, including key enzymes and TFs, was predicted and analyzed. This work may provide important insights into alkaloids biosynthesis, and reveals the regulation mechanism under precursor and MeJA treatment in *D. officinale*.

## Materials and methods

### Plant materials and experimental design

**FIGURE 1:**
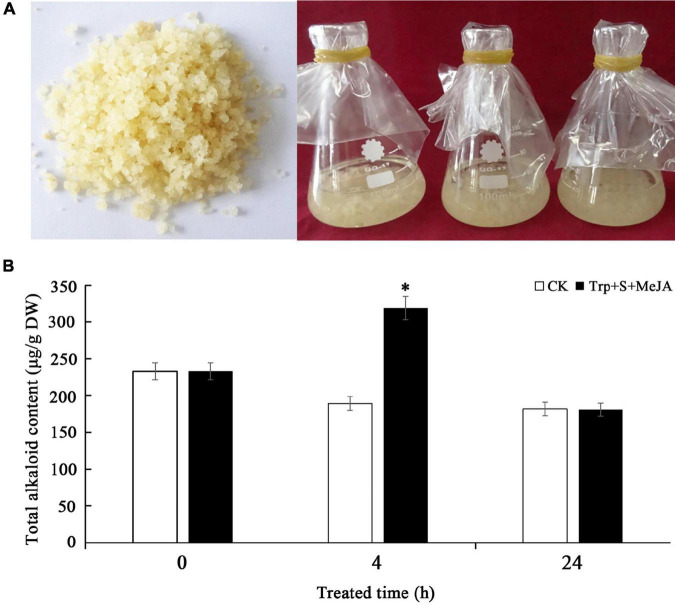
Determination of total alkaloid content in *D. officinale* PLBs under Trp + S + MeJA treatment. **(A)** The PLBs used for Trp + S + MeJA treatment. **(B)** The total alkaloid content in PLBs treated with TIA precursors and MeJA for 0, 4 and 24 h. Data represent means ± *SD* from three biological replicates. Asterisks shows significant differences based on the Student’s *t*-test (*p < 0.05).

PLBs of *D. officinale* with basically the same growth trend were used to inoculate 40 mL of 1/2 MS (Murashige and Skoog) + 0.1 mg/L NAA (α-naphthalene acetic acid) + 30 g/L sucrose + 0.1 g/L lactalbumin hydrolysate (pH 5.8) liquid medium in conical bottles, adding 7.0 g of PLBs to each bottle (Figure 1A). Based on our preliminary experimental results, 9 μmol/L Trp, 6 μmol/L secologanin (S) and 100 μmol/L MeJA were added to the liquid medium (Jiao et al., 2018). PLBs without any treatment were used as the control (CK). Samples were collected at 0, 2, 4, 8, 12, and 24 h after treatment (Ge et al., 2015; Chen et al., 2019). Sampling at each time point was performed for three biological repeats. After collection, the PLBs were cleaned under running water and divided into two groups. One group was dried at 60°C for determination of the total alkaloid content. The other group was quickly frozen with liquid nitrogen and stored at −80°C for subsequent RNA extraction and quantitative real-time polymerase chain reaction (qRT-PCR) analysis. The reagents including tryptophan, secologanin and MeJA were all purchased from Sigma-Aldrich (St. Louis, MO, United States). Other reagents, such as MS, NAA, sucrose and lactalbumin hydrolysate, were purchased from Sinopharm Chemical Reagent Co., Ltd. (Shanghai, China).

### Extraction and determination of the total alkaloid content

For alkaloid extraction, samples were soaked with ammoniated chloroform. The total alkaloid content was determined according to our previously reported method (Jiao et al., 2018).

### RNA extraction, library preparation and RNA sequencing analysis

The total RNA of *D. officinale* PLBs was extracted according to the manufacturer’s protocol for the Plant Total RNA Isolation Kit (Sangon, Shanghai, China). The concentration and quality of the extracted RNA were tested using Agilent 2100 bioanalyzer with a RNA Nano 6000 Assay Kit (Agilent, CA, United States). Four key enzyme-encoding genes that may be involved in the biosynthesis of TIAs in *D. officinale* were identified in the National Center for Biotechnology Information (NCBI) database: 1-deoxy-d-xylose 5-phosphate reductase (*DoDXS*, GenBank accession number: KF803334), strictosidine synthase (*DoSTR*, GenBank accession number: KX068707), secologanin synthase (*DoSLS*, GenBank accession number: XM_020846499.2) and tryptophan decarboxylase (*DoTDC*, GenBank accession number: MK625691.1). To determine their expression levels in PLBs at the six different treatment times mentioned above, we further selected suitable sampling points (0, 4 and 24 h) for transcriptome sequencing analysis. The qRT-PCR primers for *DoDXS*, *DoSTR*, *DoSLS* and *DoTDC* are listed in Supplementary Table 1.

Transcriptome sequencing, sequence assembly, and data analysis are provided by Biomarker Biotechnology Co., Ltd. (Beijing, China). The mRNA from these samples collected at the three sampling time points was enriched using Oligo (dT) magnetic beads. Then, the mRNA was fragmented and used as a template for reverse transcription to synthesize cDNA. The purified cDNA was then subjected to end repair and A-tailing, followed by the attachment of a sequencing adapter. A cDNA library was then obtained by PCR amplification and enrichment. Then, the sequencing was executed on an Illumina HiSeq 2,500 platform, and 150 bp paired-end reads were produced. The sequences were further processed with a bioinformatic pipeline tool, BMKCloud^13^ online platform. Raw data of fastq format were firstly processed through in-house Perl scripts. The raw data were filtered to remove the linker sequences and low-quality reads to obtain high-quality clean data. The Q20, Q30, and GC content of the clean data were calculated.

The clean data were mapped to the genome of *D. officinale* (version number ASM160598v2).^1^ HISAT2 software was used to align the obtained clean data to the reference genome, and the comparison efficiency was calculated to evaluate the assembly quality of the selected reference genome. StringTie software was used to assemble the aligned reads, and the obtained unigenes were quantitatively analyzed (Pertea et al., 2016).

### Identification and annotation of differentially expressed genes

Gene expression levels were determined using the fragments per kilobase of transcript per million mapped reads (FPKM) method (Florea et al., 2013). Differential expression analysis of two samples was performed using DESeq2 (version 1.24.0) software (Rapaport et al., 2013). The differentially expressed genes (DEGs) were screened with the threshold false discovery rate (FDR) ≤ 0.01 and | log_2_FC (fold change)| ≥ 1. Heat maps were generated using TBtools software (version 0.66837) to display genes with significantly altered expression. All the assembled unigenes and DEGs were searched against the NCBI non-redundant protein (NR),^2^ Gene Ontology (GO),^3^ Clusters of Orthologous Groups (COG),^4^ Kyoto Encyclopedia of Genes and Genomes (KEGG)^5^ and Swiss-Prot databases using a cutoff *E*-value of 10-5 (Lv et al., 2022).

### Weighted gene coexpression network analysis

A weighted gene coexpression network was built using the WGCNA R package to identify modules of high correlated genes based on the FPKM data (Langfelder and Horvath, 2008). Before performing WGCNA analysis, selected gene sets were filtered to remove low quality genes according to median absolute deviation (MAD) value. The soft thresholding power β of 14 was selected to make the networks exhibit an approximate scale-free topology (Supplementary Figure 1). The adjacency matrix was then converted to a topological overlap (TO) matrix using the TOMsimilarity algorithm (Zhan et al., 2015). All genes were hierarchically clustered based on TOMsimilarity, and a gene dendrogram was produced. The Dynamic Hybrid Tree Cut algorithm was used to cut the hierarchal clustering tree and defined modules as branches from the tree cutting. It had a module with default settings: minModuleSize was 30 and the minimum height of the combined module was 0.25 (Li et al., 2021). Modules whose eigengenes were highly correlated (correlation > 0.8) were merged. In order to screen the modules related to alkaloid biosynthesis, Pearson’s correlation between the eigengenes of each module and the alkaloid content were further analyzed. The coexpression network illustration was conducted with Cytoscape software (version 3.8.0).

### Quantitative real-time polymerase chain reaction validation

To confirm the reliability of the RNA-Seq data, the expression levels of 16 candidate genes related to alkaloid biosynthesis and metabolic regulation were checked using qRT-PCR. The primers of the 16 selected unigenes are listed in Supplementary Table 1. cDNAs were reverse-transcribed from total RNA using the PrimeScript RT Reagent Kit (Takara, Tokyo, Japan). All reactions were carried out in a QuantStudio 6 Flex real-time PCR system (Thermo Fisher, Waltham, MA, United States). Each reaction contained 2 μL of diluted cDNA, 1 μL of each primer, 8 μL of SYBR Premix Ex Taq II, and 8 μL of RNase-free double-distilled water (ddH_2_O). The cycling conditions used for qRT-PCR were as follows: 95^°^C for 3 min, followed by 40 cycles of 95^°^C for 10 s, 52^°^C for 15 s and 72^°^C for 30 s. A housekeeping gene (β-actin) was used as a reference, and the relative expression level of each gene was calculated using the 2^–Δ^ ^Δ^ *^CT^* method (Fan et al., 2016).

### Statistical analysis

Three biological replicates were prepared and analyzed in this work, and the data were presented as means ± standard deviations (SD). Student’s *t*-test and Pearson correlation analysis were performed in SPSS statistical software (version 26.0). Values of *p* < 0.05 and *p* < 0.01 were considered to be the significant differences and extremely significant differences, respectively.

## Results

### Selection of transcriptome sequencing samples

**FIGURE 2:**
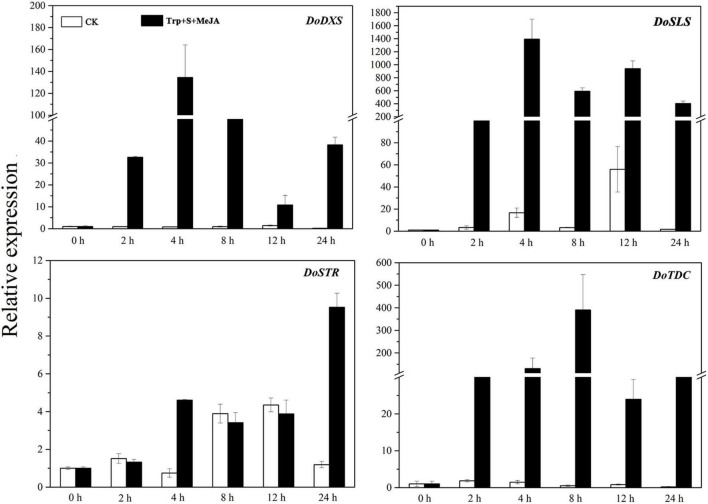
Expression patterns of four key genes involved in the TIA biosynthetic pathway of *D. officinale* PLBs under precursor and MeJA treatment.

Four structural genes, namely, *DXS*, *STR*, *SLS*, and *TDC*, are involved in the TIA biosynthetic pathway (Liu et al., 2021). To screen additional DEGs, the expression patterns of these four key enzyme-encoding genes (*DoDXS*, *DoSTR*, *DoSLS*, and *DoTDC*) in *D. officinale* PLBs were analyzed. The expression of the four genes was significantly increased under the TIA precursor and MeJA treatment (Figure 2). *DoDXS*, *DoSLS* and *DoTDC*, but not *DoSTR*, showed strong induction by the precursors and MeJA. The expression of *DoDXS* and *DoSLS* peaked after 4 h of treatment; however, *DoTDC* expression peaked after 8 h of treatment. The expression pattern of *DoSTR* was different, with a first peak observed after 4 h of treatment, followed by a second peak after 24 h. These results suggested that the expression levels of *DoDXS*, *DoSTR*, *DoSLS*, and *DoTDC* were markedly increased in the samples treated for 4 and 24 h compared with the control samples. Therefore, we selected PLBs that were treated for 0, 4 and 24 h and grew normally up to 4 and 24 h as the transcriptome sequencing samples.

### Total alkaloid content in *Dendrobium officinale* under precursor and methyl jasmonate treatment

As alkaloids are the main bioactive constituents in *D. officinale*, their levels in the control (CK) and Trp + S + MeJA-treated PLBs were measured. We observed no significant differences in total alkaloid content between PLBs treated after 24 h and non-treated samples. However, the total alkaloid content increased significantly from 189 to 319 μg/g DW after 4 h of treatment (Figure 1B). These data indicated an important role of the precursors and MeJA in the accumulation of alkaloids in *D. officinale*.

### Transcriptome sequencing analysis

To further understand the transcriptional regulatory mechanism of the precursors and MeJA in TIA biosynthesis in *D. officinale*, 15 cDNA libraries from *D. officinale* PLBs treated for different durations (0, 4 and 24 h) were constructed and sequenced. Five groups of cDNA libraries (treated after 0 h: T0_1, T0_2, T0_3; treated after 4 h: T4_1, T4_2, T4_3; treated after 24 h: T24_1, T24_2, T24_3; not treated after 4 h: C4_1, C4_2, C4_3; not treated after 24 h: C24_1, C24_2, C24_3) were separately prepared. The raw reads of the libraries were deposited in the NCBI SRA database. The quality of the RNA-Seq data is summarized in Table 1. The average Q20, Q30, and GC content were 97.30, 92.85, and 47.65%, respectively. Correlation analysis revealed that the 15 samples could be divided into five groups and that the replicates had a strong positive correlation (Supplementary Figure 2), indicating high reproducibility and reliability of the transcriptome data. In addition, 89.84% of the clean reads were mapped to the reference genome, suggesting that the selected reference genome assembly could meet the needs for information analysis.

**TABLE 1:** Summary of the sequencing quality of 15 cDNA libraries of *D. officinale*.

Sample ID	Clean reads	Clean bases (Gb)	GC content (%)	Q20 (%)	Q30 (%)	Total reads	Mapped reads (%)
T0_1	28,736,462	8.59	47.75	97.68	93.66	57,472,924	90.56
T0_2	23,934,358	7.16	47.24	97.48	93.35	47,868,716	89.66
T0_3	22,005,775	6.59	47.48	97.31	93.12	44,011,550	89.86
T4_1	25,969,819	7.76	48.37	97.21	92.66	51,939,638	89.73
T4_2	24,931,065	7.46	47.60	97.14	92.52	49,862,130	89.24
T4_3	28,620,169	8.56	48.48	96.61	91.37	57,240,338	89.52
T24_1	26,728,413	7.99	48.22	97.34	92.91	53,456,826	90.14
T24_2	31,013,546	9.27	48.51	97.55	93.41	62,027,092	90.66
T24_3	36,684,049	10.96	47.80	97.43	93.14	73,368,098	89.95
C4_1	73,368,098	8.29	46.77	97.63	93.47	55,486,642	89.55
C4_2	62,038,475	18.54	47.26	97.30	92.76	124,076,950	89.95
C4_3	28,981,610	8.65	47.18	97.25	92.67	57,963,220	89.56
C24_1	28,584,908	8.54	47.32	97.18	92.46	57,169,816	89.94
C24_2	35,182,090	10.51	47.55	97.24	92.69	70,364,180	89.78
C24_3	29,379,576	8.77	47.15	97.18	92.58	58,759,152	89.71
Average	30,702,242	9.18	47.65	97.30	92.85	61,404,485	89.84

### Functional annotation and classification of unigenes

A total of 32,727 unigenes were obtained, of which 29,149 were from the genome of *D. officinale* and 3,578 were new genes. These unigenes were annotated in public databases, including the NR, GO, COG, Swiss-Prot, and KEGG databases. Among them, 31,979 unigenes were annotated in at least one database.

GO classification was used to classify unigene functions based on the NR annotation. Of the 31,797 assembled unigenes, 12,078 unigenes were successfully assigned to one or more GO terms and classified into three main GO categories and 50 groups (Supplementary Figure 3A). Within the “biological process” (BP) domain, the most evident matches were in the terms “metabolic process” (5,783 unigenes), “cellular process” (5,276 unigenes), and “single-organism process” (3,535 unigenes). In the “cellular component” (CC) domain, most of the unigenes were assigned to the terms “cell” (5,376 unigenes) and “cell part” (5,355 unigenes). In the “molecular function” (MF) domain, the assignments were mostly enriched in the term “catalytic activity” (5,769 unigenes).

All the unigenes were also subjected to a search against the COG database for functional product information and classification. A total of 9,047 unigenes were assigned a COG functional classification and divided into 25 categories (Supplementary Figure 3B). Among them, the most common group was “general function prediction only” (1,003 unigenes, 11.09%), followed by “signal transduction mechanisms” (906 unigenes, 10.01%) and “translation, ribosomal structure and biogenesis” (870 unigenes, 9.62%). Notably, 525 unigenes were annotated in the “secondary metabolites biosynthesis, transport and catabolism” group, suggesting that these unigenes may be related to alkaloid biosynthesis in *D. officinale* PLBs.

Then, KEGG pathway analysis was performed to functionally classify biochemical pathways associated with the unigenes. A total of 4,783 unigenes were assigned to five KEGG categories with 120 subcategories: “metabolism,” “genetic information processing,” “environmental information processing,” “cellular processes” and “organismal systems” (Supplementary Figure 4). A summary of the findings is presented in Supplementary Table 2. Of the KEGG secondary metabolic pathways, 311 unigenes were assigned to “flavonoid biosynthesis,” “flavone and flavonol biosynthesis,” “phenylpropanoid biosynthesis,” “isoquinoline alkaloid biosynthesis” and “tropane, piperidine and pyridine alkaloid biosynthesis.” The metabolic pathways annotated in the KEGG database, such as “phenylalanine, tyrosine and tryptophan biosynthesis” (ko00400) and “terpenoid backbone biosynthesis” (ko00900), were related to the TIA biosynthesis pathway.

### Identification and functional annotation of the differentially expressed genes between the control and treated protocorm-like bodies in *Dendrobium officinale*

**FIGURE 3:**
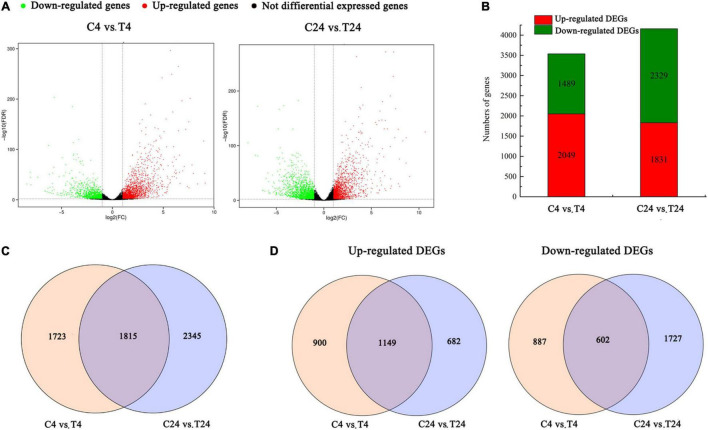
Differentially expressed genes (DEGs) identified by RNA-Seq analysis in Trp + S + MeJA-treated and control samples after induction for 4 and 24 h. **(A)** Volcano plot of the RNA-Seq data showing DEGs in red and green. The *x*-axis indicates the fold change in C4 vs. T4 and C24 vs. T24. The *y*-axis represents the negative -log_10_-transformed *p-*values (FDR < 0.01) for differences between the samples. **(B)** DEG numbers in the C4 vs. T4 and C24 vs. 24 groups. **(C)** Venn diagram of all DEGs in the C4 vs. T4 and C24 vs. T24 groups. **(D)** Venn diagram of upregulated and downregulated DEGs in the C4 vs. T4 and C24 vs. T24 groups.

To identify the differentially expressed unigenes between these samples, we used FDR < 0.01 and | log_2_FC (fold change)| ≥ 1 as the selection parameters. Here, the comparison of C4 vs. T4 and C24 vs. T24 yielded 3,538 and 4,160 DEGs, respectively. A volcano plot was constructed to illustrate the distribution of DEGs in these two comparison groups (Figure 3A). Among the detected DEGs, the C4 vs. T4 group had 2,049 upregulated and 1,489 downregulated genes, and the C24 vs. T24 group had 1,831 upregulated and 2,329 downregulated genes (Figure 3B). In particular, 1,815 DEGs were present in both comparison groups, 1,723 DEGs were present in only the C4 vs. T4 group, and 2,345 DEGs were present in only the C24 vs. T24 group (Figure 3C). In addition, among the 2,731 upregulated DEGs, 1,149 were shared by the two comparison groups, 900 were present in only the C4 vs. T4 group, and 682 DEGs were present in only the C24 vs. T24 group. There were 3,216 downregulated DEGs in the two comparison groups, among which 602 were present in both comparison groups, 887 were present in only the C4 vs. T4 group, and 1,727 DEGs were present in only the C24 vs. T24 group (Figure 3D).

**FIGURE 4:**
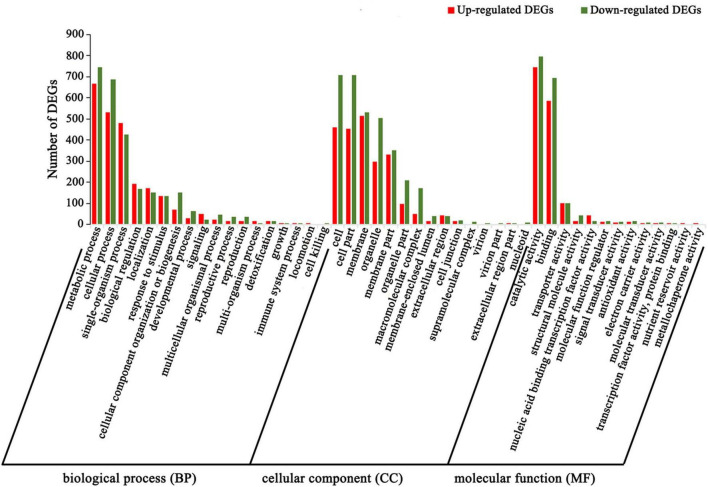
GO enrichment of DEGs in Trp + S + MeJA-treated and non-treated PLBs of *D. officinale*. The *x*-axis indicates the percentage of DEGs in the subcategories of each main category. The *y*-axis indicates the number of DEGs in each subcategory.

**FIGURE 5:**
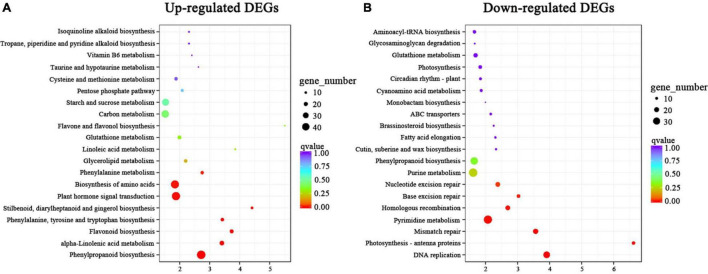
KEGG enrichment analysis of DEGs in Trp + S + MeJA-treated and non-treated PLBs of *D. officinale*. **(A)** KEGG enrichment analysis of upregulated DEGs. **(B)** KEGG enrichment analysis of downregulated DEGs. The *y*-axis and *x*-axis represent the KEGG pathways and the enrichment factors, respectively. The greater the enrichment factor is, the more obvious the enrichment level of DEGs in the pathway. The color of the block represents the *q*-value, and the *q*-value is the *p-*value corrected by multiple hypothesis tests. The smaller the *q*-value is, the more reliable the enrichment of DEGs in this pathway is. The size of the circle indicates the number of genes enriched in the pathway.

To further understand the biological functions of the DEGs, we compared these DEGs against the GO and KEGG databases. The 2,731 upregulated and 3,216 downregulated DEGs in the above two comparison groups (C4 vs. T4 and C24 vs. T24) were analyzed by GO and KEGG enrichment analysis. In the GO analysis, the “single-organism process,” “biological regulation,” “localization,” “signaling,” “multi-organism process,” “transporter activity,” and “nucleic acid binding transcription factor activity” subcategories contained mainly upregulated DEGs, whereas the other subcategories contained mainly downregulated DEGs (Figure 4). In the KEGG analysis, upregulated DEGs were mainly enriched in alkaloid biosynthesis pathways, including “isoquinoline alkaloid biosynthesis,” “tropane, piperidine and pyridine alkaloid biosynthesis,” and “phenylalanine, tyrosine and tryptophan biosynthesis” (Figure 5A). Some upregulated DEGs were also significantly enriched in “plant hormone signal transduction” and “α-linolenic acid metabolism” (endogenous JA biosynthesis pathway). The downregulated DEGs were mainly related to plant growth and development, fatty acid metabolism, and amino acid and energy metabolism pathways, such as “photosynthesis-antenna proteins,” “carbon fixation in photosynthetic organisms,” “pyrimidine metabolism” and “cutin, suberine and wax biosynthesis” (Figure 5B).

### Identification of modules associated with alkaloid biosynthesis in *Dendrobium officinale* protocorm-like bodies by weighted gene coexpression network analysis

**FIGURE 6:**
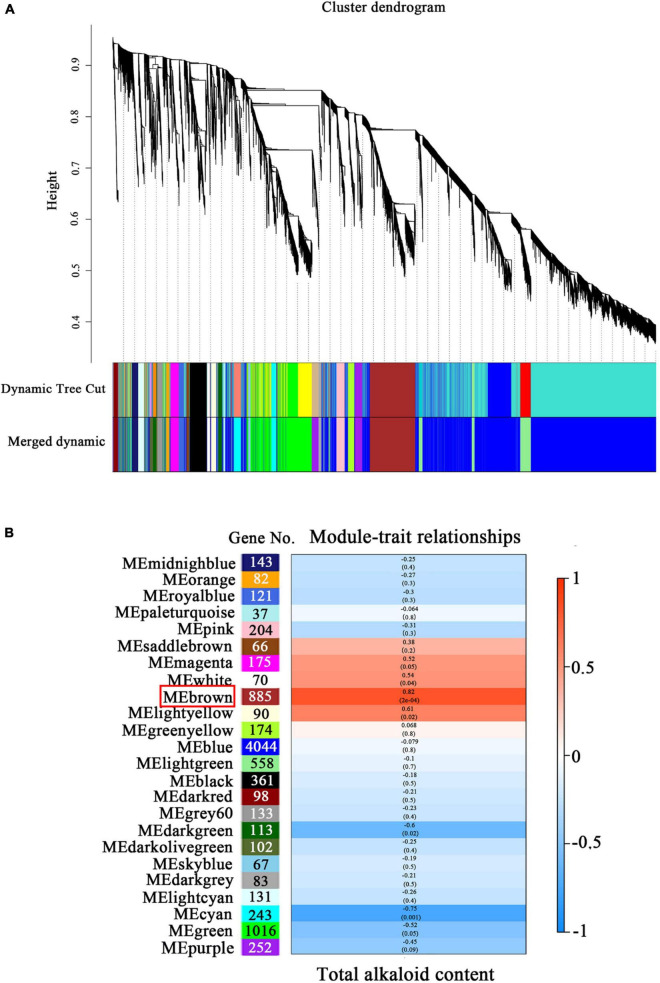
Weighted gene coexpression network analysis (WGCNA) and modules related to alkaloid biosynthesis in *D. officinale* PLBs. **(A)** Dendrogram with color annotation. **(B)** Pearson correlation coefficient (*r*) and the *p*-value for alkaloid content and each module. The color scale on the right shows module-trait correlation from −1 (blue) to 1 (red).

To identify the genes associated with alkaloid biosynthesis under precursor and MeJA treatment, a WGCNA was performed with all 9,248 genes, resulting in 24 coexpressed modules (Figure 6A). The largest module (“blue”) contained 4,044 genes, while the smallest module (“paleturquoise”) contained 37 genes (Figure 6B). Subsequently, the total alkaloid content was used as a phenotype for correlation analysis with the obtained modules. The “brown” module contained 885 genes was significantly positively correlated with alkaloid, with correlation coefficient (*r*) of 0.82 (*p* = 2 × 10^–4^).

Based on the functional annotation and WGCNA results, the genes involved in the TIA biosynthesis [mevalonate (MVA) pathway, 2-C-methyl-D-erythritol 4-phosphate (MEP), seco-iridoid pathway, shikimate pathway and TIA downstream biosynthetic pathway], isoquinoline alkaloid biosynthesis, tropane alkaloid biosynthesis and JA biosynthesis (α-linolenic acid metabolism) were further analyzed. Finally, we identified 125 unigenes annotated as 36 enzymes involved in alkaloid biosynthesis (Supplementary Table 3).

**FIGURE 7:**
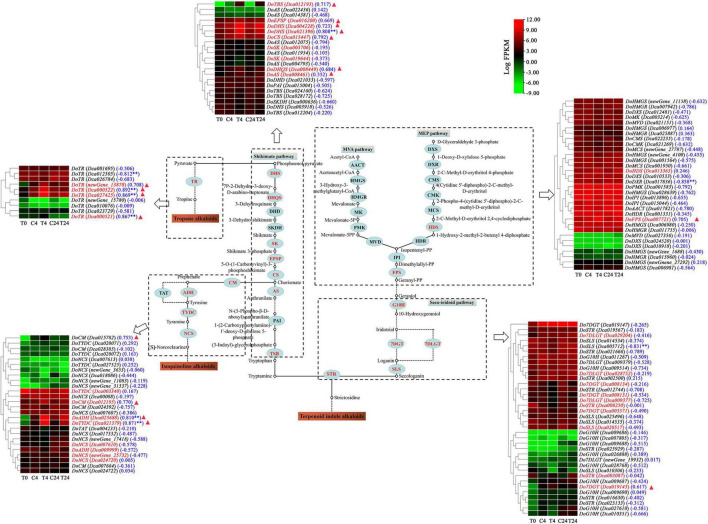
Expression patterns of genes putatively involved in the biosynthesis of alkaloids in *D. officinale* PLBs under precursor and MeJA treatment. The enzymes are marked in red, indicating that genes encoding the enzymes showed different expressions in the C4 vs. T4 or C24 vs. T24 groups. The genes marked in red in the heat map indicates the DEGs identified from the C4 vs. T4 or C24 vs. T24 groups. Blue numbers in brackets represent the correlation coefficient which analyzed between alkaloid content and biosynthesis-related genes. ^**^p < 0.01 and —*r*— > 0.8. The red triangle indicates that the gene was found in the brown module.

Furthermore, the transcriptomic analysis showed that 35 unigenes exhibited differential expression, and most of them were highly expressed in the Trp + S + MeJA-treated groups (4 and 24 h treated PLBs) compared to the non-treated groups (Figure 7). Of these DEGs, 23 were related to TIA biosynthesis, and 15 showed upregulation under Trp + S + MeJA treatment after 4 or 24 h; these DEGs encode putative HDS (Dca013365), FPS (Dca007721), G10H (Dca009687 and Dca027610), 7DGT (Dca019145), 7DLGT (Dca028752 and Dca029204), DHS (Dca004228 and Dca021396), DHQS (Dca008449), EPSP (Dca016200), CS (Dca015447), AS (Dca008461), TBS (Dca012193) and STR (Dca003087) proteins. However, the expression of *DoSLS* and *DoSK*, important genes in the seco-iridoid and shikimate pathways, respectively, decreased after Trp + S + MeJA treatment. Twelve DEGs were identified as being involved in isoquinoline and tropane alkaloid biosynthesis, all of which were upregulated by Trp + S + MeJA treatment except *DoADH* (*Dca009999*). The increased expression of these genes subsequently induced alkaloid accumulation, suggesting that the precursors and MeJA could stimulate secondary metabolism via the main biosynthesis pathways and enhance the alkaloid content of PLBs in *D. officinale.*

**FIGURE 8:**
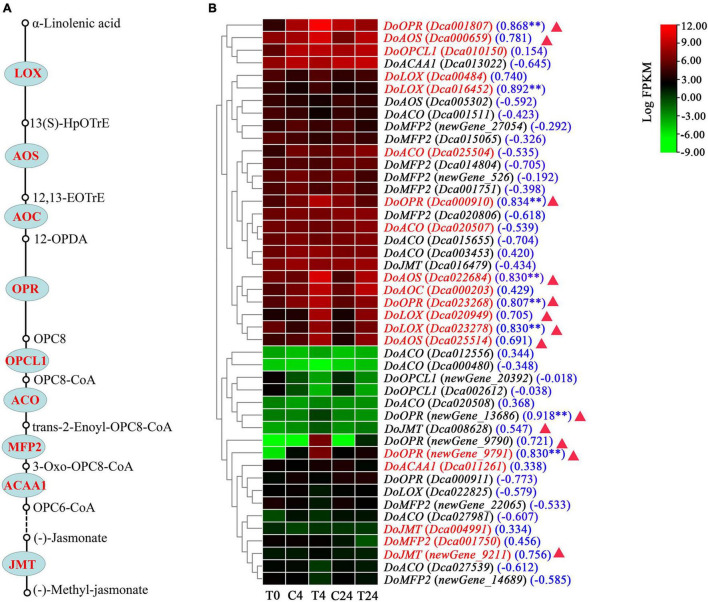
Expression pattern analysis of genes involved in JA biosynthesis in *D. officinale* PLBs under the precursor and MeJA treatment. **(A)** JA biosynthesis pathway. The enzymes are marked in red, indicating that genes encoding the enzymes were differentially expressed in the C4 vs. T4 or C24 vs. T24 groups. **(B)** Heat map of genes associated with JA biosynthesis. The genes marked in red in the heat map indicates the DEGs identified from the C4 vs. T4 or C24 vs. T24 groups. Blue numbers in brackets represent the correlation coefficient which analyzed between alkaloid content and JA biosynthesis-related genes. ^**^p < 0.01 and —*r*— > 0.8. The red triangle indicates that the gene was found in the brown module.

JA derivatives, including jasmonoyl-isoleeucine (JA-ile) and MeJA, are known to promote alkaloid accumulation in various plants (Wasternack and Strnad, 2019). Therefore, we next analyzed the key genes in the JA biosynthesis pathway. As shown in Figure 8A and Supplementary Table 4, we identified 45 unigenes that encoded nine enzymes involved in the biosynthetic pathway of JA. The majority of the genes functioning in this pathway were upregulated, with 18 DEGs in the C4 vs. T4 or C24 vs. T24 group (Figure 8B). Further analysis showed that their expression patterns were similar to those of the DEGs in the alkaloid biosynthesis pathway at different treatment times. Strikingly, the differential expression was more significant at 4 h than at 24 h after Trp + S + MeJA treatment. For example, 29.45- and 1625.08-fold increases in *DoLOX* (*Dca020949*) and *DoOPR* (*newGene_9791*) expression were observed after 4 h of treatment, but the expression levels increased only 14.56- and 158.31-fold, respectively, at 24 h.

A correlation analysis between alkaloid content and the transcriptional abundance of key genes involving the TIA, isoquinoline alkaloid, tropane alkaloid and JA biosynthesis pathways was further conducted. As shown in Figures 7, 8B, the alkaloid content was highly correlated with the expression of nine and eight genes associated with alkaloid biosynthetic pathway and JA biosynthesis pathway, respectively, (|*r*| > 0.8 and *p* < 0.01). In particular, six genes involved in alkaloid biosynthesis, such as *DHS* (*Dca021396*), *DoADH* (*Dca025688*), *DoTYDC* (*Dca021379*), and *DoTR* (*Dca000522*, *Dca027425*, and *Dca000521*), and seven genes related to JA bisynthesis, including *DoLOX* (*Dca023278*), *DoAOS* (*Dca022684*) and *DoOPR* (*newGene_9791*, *newGene_13686*, *Dca023268*, *Dca001807*, and *Dca000910*) were also found in the brown module. The above results indicated that these genes were very relevant to alkaloid biosynthesis in *D. officinale*.

### Transcription factors related to alkaloid biosynthesis in *Dendrobium officinale* protocorm-like bodies

Various TFs have been reported to participate in the biosynthesis of secondary metabolites in plants. In our study, 1,438 unigenes belonging to 48 major TF families were identified in *D. officinale* PLBs (Supplementary Table 5). Members of the MYB, C2H2, AP2/ERF, NAC, and bHLH families constituted the top five classes, each with more than 80 unigenes, and the MYB family was the largest family, containing 170 members. However, members of the CSD, LIM, Whirly and LFY families had < 4 unigenes.

**FIGURE 9:**
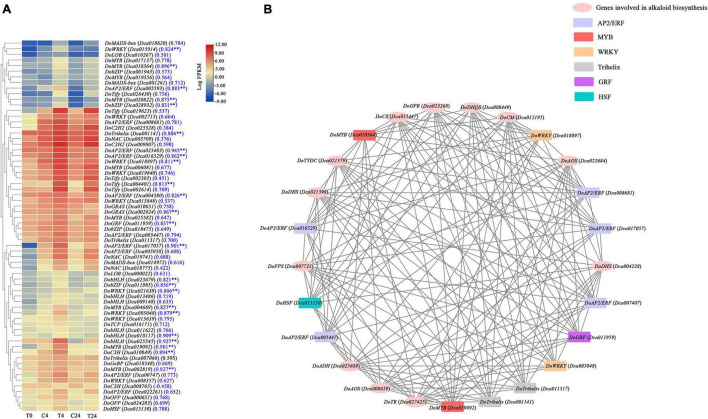
Coexpression network of TFs and structure genes related to alkaloid biosynthesis pathway in *D. officinale* PLBs under the precursor and MeJA treatment. **(A)** Heat map displaying the expression of 66 TFs in different samples. **(B)** Construction of regulatory networks of TFs and structure genes related to alkaloid biosynthesis. **p < 0.01 and |*r*| > 0.8.

To systematically study the regulatory networks of alkaloid biosynthesis-related genes and TFs, a total of 66 TFs were found in the brown module, and their expression patterns are presented in Figure 9A. Most of these TFs exhibited similar expression patterns; their expression levels were significantly upregulated at 4 h and remained high at 24 h. As shown in Figure 9A, these TFs belonged to 18 TF families, including AP2/ERF (10), MYB (9), WRKY (9), bHLH (6), Tify (5), bZIP (4), NAC (3), Trihelix (3), MADS-box (3), C3H (2), OFP (2), GRAS (2), C2H2 (2), LOB (2), HSF (1),TCP (1), GRF (1), and GeBP (1). Among them, the expression of 24 TFs were positively correlated with the total alkaloid content (*r* > 0.8 and *p* < 0.01). The interaction network diagram analyzed with Cytoscape showed that 13 of the above-mentioned TFs were highly coexpressed with *DoFPS* (*Dca007721*), *DoDHQS* (*Dca008449*), *DoDHS* (*Dca021396* and *Dca004228*), *DoCS* (*Dca015447*), *DoCM* (*Dca012195*), *DoTYDC* (*Dca021379*), *DoADH* (*Dca025688*), *DoTR* (*Dca027425*), *DoAOS* (*Dca022684* and *Dca000659*), and *DoOPR* (*Dca023268*) (Figure 9B), which further illustrated that they play a vital role in the regulation of alkaloid biosynthesis in *D. officinale*.

### Quantitative real-time polymerase chain reaction validation analysis of differentially expressed genes

**FIGURE 10:**
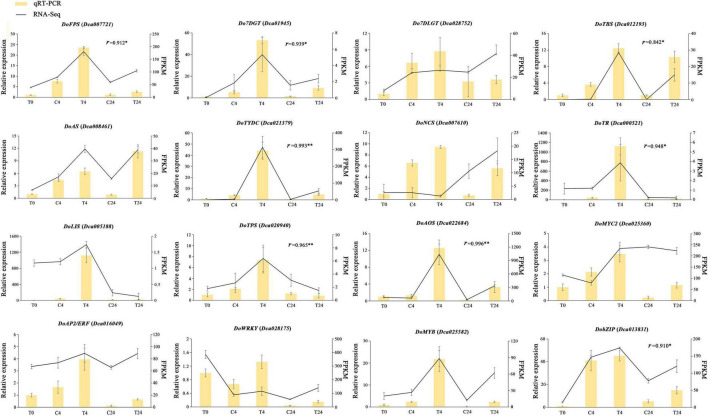
qRT-PCR validation of DEGs related to alkaloid biosynthesis and metabolic regulation in *D. officinale* PLBs. The left *y*-axis represents the relative expression levels corresponding to the histogram. The right *y*-axis represents expression levels calculated by the FPKM method corresponding to the line plots. The relationship between the RNA-Seq and qRT-PCR in T0, C4, T4, C24, and T24 samples was analyzed based on Pearson’s correlation analysis and *r* represents correlation coefficients. *p < 0.05, ^**^p < 0.01.

To verify the accuracy of the transcriptomic data, the expression levels of five DEGs related to TIA biosynthesis: *DoFPS* (*Dca007721*), *Do7DGT* (*Dca019145*), *Do7DLGT* (*Dca028752*), *DoTBS* (*Dca012193*), and *DoAS* (*Dca008461*); two DEGs related to isoquinoline alkaloid: *DoTYDC* (*Dca021379*) and *DoNCS* (*Dca007610*); one DEG related to tropane alkaloid: *DoTR* (*Dca000521*); two terpene synthase DEGs: *DoLIS* (*Dca005188*) and *DoTPS* (*Dca020940*); one DEG involved in JA biosynthesis: *DoAOS* (*Dca022684*); and five TFs regulating alkaloid biosynthesis: *DoMYC2* (*Dca025360*), *DoAP2/ERF* (*Dca016049*), *DoWRKY* (*Dca028175*), *DoMYB* (*Dca025582)* and *DobZIP* (*Dca013831*) were checked using qRT-PCR, with three biological replicates tested. The relationship between the RNA-Seq and qRT-PCR in different samples was also analyzed based on Pearson’s correlation analysis. Results showed that the relative expression trend of most genes was basically consistent with the RNA-Seq data, and some correlation values were higher than 0.8 (Figure 10), which further demonstrated the reliability of the transcriptome data. These results confirmed that our transcriptome data could serve as a foundation for further research on related genes associated with other medicinal components of *D. officinale*.

## Discussion

### Identification of genes involved in the biosynthetic pathways of three types of alkaloids

*D. officinale* is well known as one of the most important traditional herbs in China, and its alkaloids are its main medicinal components. However, the regulatory mechanism underlying the precursor and MeJA-induced accumulation of alkaloids in *D. officinale* PLBs is still unknown. To date, a number of transcriptomic analyses have been performed for different reasons, such as the identification of genes associated with the biosynthesis of bioactive components (polysaccharides, flavonoids, alkaloids, and terpenes) (Ren et al., 2020; Wang et al., 2020; Li et al., 2021), flower development (He et al., 2020; Chen et al., 2021), and environmental responses (Jiang et al., 2020; Zhang et al., 2021). In this study, *D. officinale* PLBs treated with TIA precursors (Trp and S) and MeJA were used for transcriptome sequencing on the Illumina HiSeq platform. Genes associated with the biosynthesis of three types of alkaloids, namely, TIAs, isoquinoline alkaloids and tropane alkaloids, were annotated in the transcriptomic data. This result was also consistent with our metabonomic analysis (Jiao et al., 2018), which further confirmed that these three types of alkaloids exist in *D. officinale* PLBs.

## Precursors and methyl jasmonate induce the transcription of genes related to alkaloid biosynthesis in *Dendrobium officinale* protocorm-like bodies

Generally, precursors and MeJA can regulate the expression of biosynthetic genes in the TIA pathway (Wei, 2010; Pan et al., 2018; Sharma et al., 2019). The transcriptomes of *D. officinale* PLBs treated with TIA precursors and MeJA for 4 and 24 h were compared with those of PLBs grown normally for 4 and 24 h, respectively. We found that the TIA precursors and MeJA could positively regulate the transcription of most genes of the TIA, isoquinoline alkaloid and tropane alkaloid biosynthesis pathways to promote the accumulation of alkaloids. Genes in the MVA/MEP pathway (*DoHDS* and *DoFPS*), seco-iridoid pathway (*DoG10H, Do7DGT*, and *Do7DLGT*), shikimate pathway (*DoDHS*, *DoDHQS, DoEPSP*, *DoCS*, *DoAS*, and *DoTBS*) and a key enzyme-encoding gene (*DoSTR*) were all upregulated significantly (Figure 7). Therefore, it is speculated that the precursors and MeJA promote the accumulation of TIAs by regulating the transcription of genes related to the TIA biosynthesis pathway in *Dendrobium officinale* PLBs.

A similar phenomenon was also observed for isoquinoline and tropane alkaloid biosynthetic genes, including *DoADH*, *DoCM*, *DoTYDC*, and *DoNCS* in the isoquinoline alkaloid biosynthetic pathway and *DoTR* in the tropane alkaloid biosynthetic pathway, which showed a significant upward trend at the transcriptional level. As reported previously, transcriptome analysis of *Papaver somniferum* treated with MeJA showed that the expression of *PsNCS*, which belongs to the isoquinoline alkaloid biosynthesis pathway, was significantly upregulated (Gurkok et al., 2015). This result is consistent with our study. Thus, we speculate that precursors and MeJA can promote the biosynthesis of isoquinoline alkaloids (such as xanthoplanine, which was identified in our previous study) by positively regulating the expression levels of *DoADH*, *DoCM*, *DoTYDC*, and *DoNCS*. Tropinone reductase (TR), a stereospecific NADPH-dependent reductase, is a key enzyme involved in the metabolism of tropinone (Kohnen-Johannsen and Kayser, 2019). TR has been found in *Dendrobium huoshanense* and *Dendrobium nobile*, and its expression was also induced by MeJA (Wang et al., 2020). In our study, the expression of *DoTR* was significantly upregulated after Trp + S + MeJA treatment (Figure 7). This indicated that *DoTR* was involved in regulating the biosynthesis of tigloidine (a type of tropane alkaloid found in our previous study) in *D. officinale* PLBs.

Exogenous MeJA can induce the expression of genes related to the endogenous JA biosynthesis pathway to improve the level of endogenous JA in plants, which constitutes the self-activation of JA biosynthesis (Wasternack and Strnad, 2019; Griffiths, 2020). Here, our transcriptomic analysis showed that the expression levels of endogenous JA biosynthesis pathway-related genes, such as *DoLOX*, *DoAOS*, *DoOPR*, and *DoJMT*, increased significantly in Trp + S + MeJA-treated *D. officinale* PLBs. It is speculated that treatment with TIA precursors and MeJA leads to the upregulation of the transcription of genes related to the JA biosynthesis pathway to improve the expression of α-linolenic acid and the endogenous JA levels (Jiao et al., 2018).

WGCNA was used to analyze the correlation between physiological indicators and genes (Yang et al., 2021). This method has been applied to study the regulatory networks of the triterpene saponin biosynthesis in tea (*Camellia sinensis*) (Chen et al., 2022), anthocyanin accumulation in apple (*Malus domestica*) (Song et al., 2019), and lignin metabolism in pomelo (*Citrus maxima*) (Li et al., 2022). In our study, WGCNA was also used to further analyze the association between the coexpressed gene modules formed by all genes and the alkaloid content. The brown module was highly positively correlated (0.82) with the alkaloid content and contained 885 genes, indicating these genes might play an important role in alkaloid biosynthesis (Figure 6B). In the brown module, a total of six genes involved in alkaloid biosynthesis, such as *DoDHS*, *DoADH*, *DoTYDC*, and *DoTR*, and seven genes related to JA bisynthesis, such as *DoLOX*, *DoAOS*, and *DoOPR*, were significantly correlated with the alkaloid content and may play a vital role in the alkaloid biosynthesis of *D. officinale*.

### Transcription factors are involved in regulating the alkaloid biosynthesis in *Dendrobium officinale* protocorm-like bodies

As reported previously, the TFs involved in regulating the biosynthesis of TIAs or isoquinoline alkaloids are mainly bHLH, AP2/ERF, WRKY, MYB, and bZIP family members (Zhou and Memelink, 2016). In *C. roseus*, it has been suggested that CrMYC1 and CrMYC2 (bHLH TFs), CrORCA1, CrORCA2, and CrORCA3 (AP2/ERF TFs), CrWRKY1 (a WRKY TF), CrBPF1 (an MYB TF), and CrGBF1, and CrGBF2 (bZIP TFs) can regulate the expression of genes related to the TIA biosynthesis pathway (Sibéril et al., 2001; Zhou and Memelink, 2016). Furthermore, CjWRKY1 and CjbHLH1 TFs, which can regulate the biosynthesis of isoquinoline alkaloids, have been found in *Coptis japonica* (Kato et al., 2007). Besides, JAZ proteins, belonging to the plant-specific Tify family, also play important regulatory role in alkaloids biosynthesis (Yamada and Sato, 2021). In this study, a total of 66 TFs, which mostly belonged to AP2/ERF, MYB, bHLH, WRKY, bZIP, and Tify families, were obtained after WGCNA analysis, and their expression were almost positively related to the alkaloid content. Moreover, 13 of the above TFs might control alkaloid biosynthesis by transcriptionally regulating *DoFPS* (*Dca007721*), *DoDHQS* (*Dca008449*), *DoDHS* (*Dca021396* and *Dca004228*), *DoCS* (*Dca015447*), *DoCM* (*Dca012195*), *DoTYDC* (*Dca021379*), *DoADH* (*Dca025688*), *DoTR* (*Dca027425*), *DoAOS* (*Dca022684* and *Dca000659*), and *DoOPR* (*Dca023268*) (Figure 9B). So far, the knowledge on the TFs to alkaloid biosynthesis in *D. officinale* are still limited. More studies are needed to further explore the regulatory network of alkaloid metabolism in *D. officinale*.

## Conclusion

In this study, we compared the total alkaloid content in treated (Trp + S + MeJA) and non-treated *D. officinale* PLBs, and a comparative transcriptomic analysis was performed. The total alkaloid content in 4 h-treated PLBs was much higher than that in 4 h-non-treated samples. 13 genes encoding DoDHS, DoADH, DoTYDC, DoTR, DoLOX, DoAOS, and DoOPR enzymes, might be the key genes involved in alkaloid biosynthesis. In addition, most of the 13 TFs belonging to AP2/ERF, WRKY, and MYB gene families, were predicted to be associated with alkaloid accumulation by WGCNA, and deserve to be further investigated as key regulators of alkaloid metabolism. This study will facilitate future research on transcriptional regulation mechanisms in *D. officinale* and other traditional Chinese medicinal plants.

## Data availability statement

Our RNA-seq data of 15 cDNA libraries have been deposited in the repositories of NCBI Sequence Read Archive with Bioproject accession number: PRJNA816308 and SRA accession numbers: SRR18338800-SRR18338814.

## Author contributions

QJ, HF, and YC conceived the study and participated in its design and coordination. CJ performed the experimental measurements, processed the experimental data, and wrote the manuscript. MW and CS interpreted the experimental data. ZW and CS helped in revising the manuscript and pictures. All authors contributed to the article and approved the submitted version.

## Conflict of interest

The authors declare that the research was conducted in the absence of any commercial or financial relationships that could be construed as a potential conflict of interest. The handling editor, YC, declared a past co-authorship with the authors, YC and CS.

## Publisher’s note

All claims expressed in this article are solely those of the authors and do not necessarily represent those of their affiliated organizations, or those of the publisher, the editors and the reviewers. Any product that may be evaluated in this article, or claim that may be made by its manufacturer, is not guaranteed or endorsed by the publisher.

## Footnotes

1
https://www.ncbi.nlm.nih.gov/assembly/GCF_001605985.2/

2
http://www.nlm.nih.gov

3
http://www.geneontology.org/

4
http://www.ncbi.nlm.nih.gov

5
http://www.genome.jp/kegg/
