# Supplementary material for: Transcriptomic analysis of genes related to alkaloid biosynthesis and the regulation mechanism under precursor and methyl jasmonate treatment in Dendrobium officinale
Source: Front Plant Sci. 2022 Jul 22;13:941231. doi: 10.3389/fpls.2022.941231 (PMC9355482; doi:10.3389/fpls.2022.941231)
Supplement: Supplementary file 1 [file Image_1.pdf]

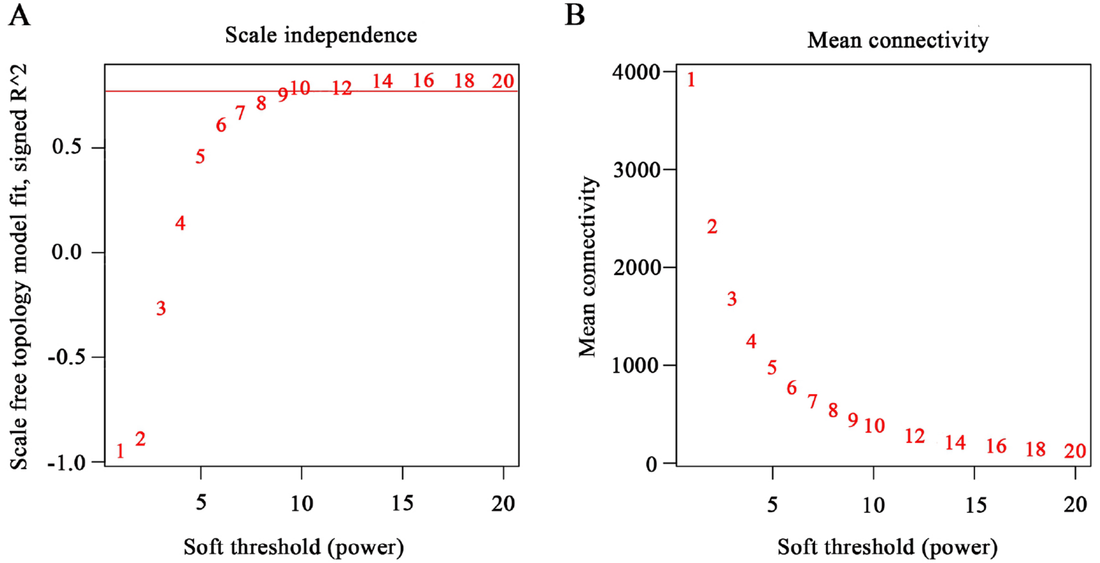

Supplementary Figure 1. Determination of soft-thresholding power. (**A**) Analysis of the scale-free fit index for various soft-thresholding powers (β). (**B**) Analysis of the mean connectivity for various soft-thresholding powers (β).
