# Supplementary material for: Transcriptomic analysis of genes related to alkaloid biosynthesis and the regulation mechanism under precursor and methyl jasmonate treatment in Dendrobium officinale
Source: Front Plant Sci. 2022 Jul 22;13:941231. doi: 10.3389/fpls.2022.941231 (PMC9355482; doi:10.3389/fpls.2022.941231)
Supplement: Supplementary file 2 [file Image_2.pdf]

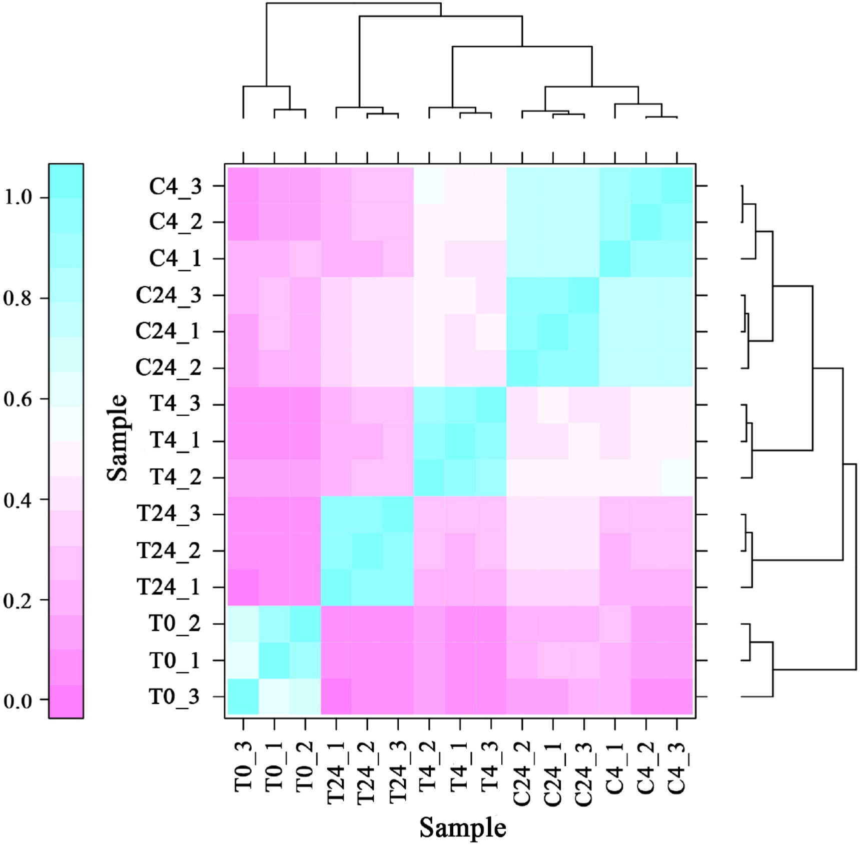

Supplementary Figure 2. The correlation heat map of samples selected for comparative transcriptome analysis. The left gradient barcode color indicates the minimum value (0) in lavender and the maximum (1) in light blue. A value close to 1 indicates a high positive correlation, while a value close to 0 means no correlation.
