# Supplementary material for: Transcriptomic analysis of genes related to alkaloid biosynthesis and the regulation mechanism under precursor and methyl jasmonate treatment in Dendrobium officinale
Source: Front Plant Sci. 2022 Jul 22;13:941231. doi: 10.3389/fpls.2022.941231 (PMC9355482; doi:10.3389/fpls.2022.941231)
Supplement: Supplementary file 4 [file Image_4.pdf]

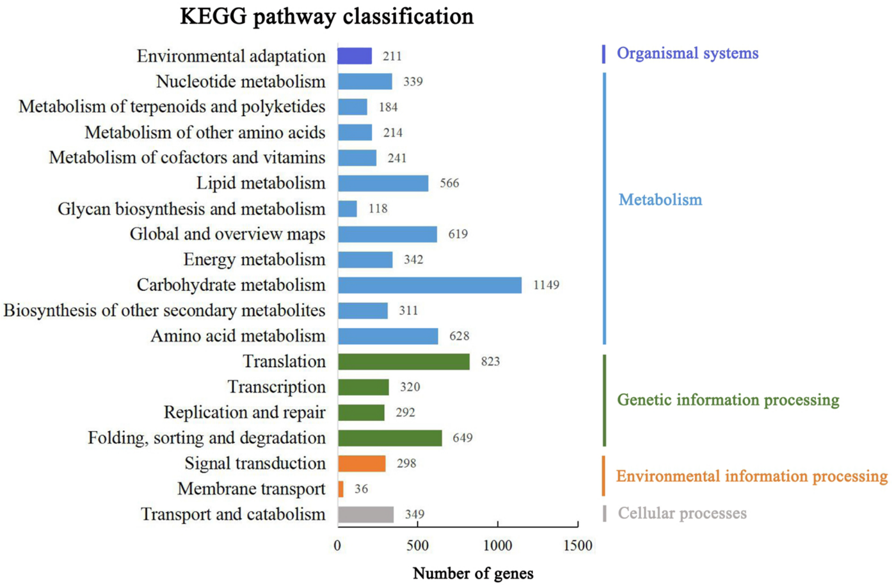

Supplementary Figure 4. Functional classification and pathway assignment of unigenes by KEGG in *D. officinale* PLBs. The unigenes were classified into five main categories in KEGG classification. The *x*-axis indicates the number of unigenes in category, and the *y*-axis represents the name of KEGG metabolic pathway.
