# Supplementary material for: Transcriptomic analysis of genes related to alkaloid biosynthesis and the regulation mechanism under precursor and methyl jasmonate treatment in Dendrobium officinale
Source: Front Plant Sci. 2022 Jul 22;13:941231. doi: 10.3389/fpls.2022.941231 (PMC9355482; doi:10.3389/fpls.2022.941231)
Supplement: Supplementary file 3 [file Image_3.pdf]

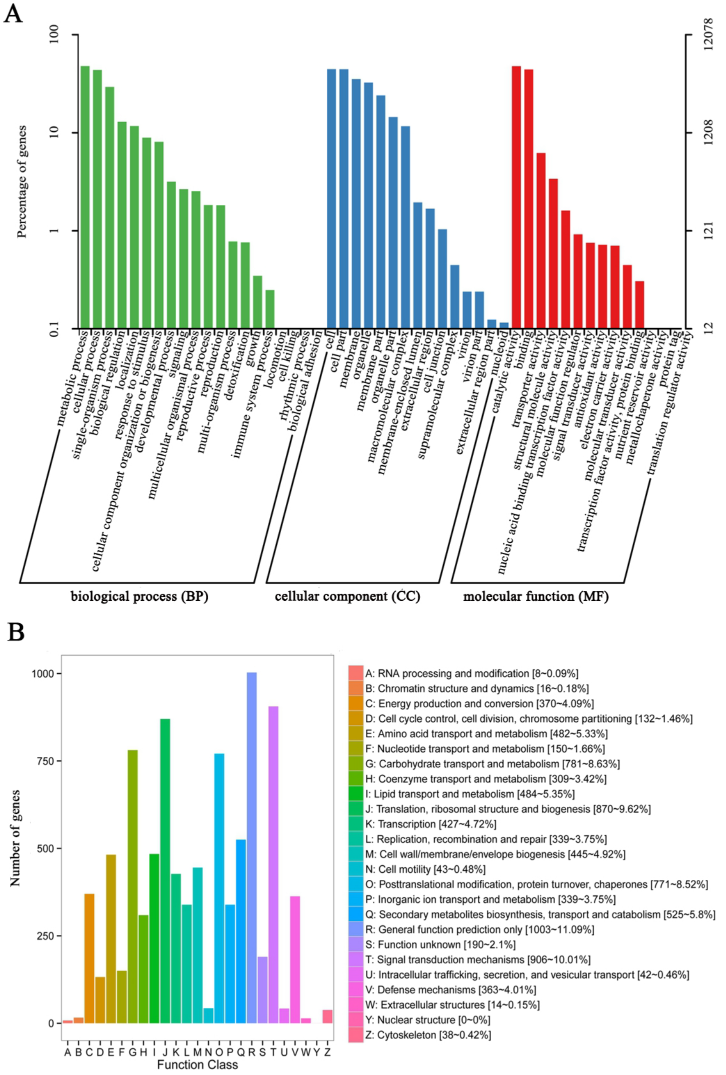

Supplementary Figure 3. GO and COG classification of unigenes in *D. officinale* PLBs. (**A**) GO classification. A total of 12,078 unigenes were classified into three main categories and 50 groups, 20 groups in "biological process" domain, 15 in "cellular component" domain, and 15 in "molecular function" domain, respectively. The left-hand and right-hand *y*-axis respectively represent the percentage of a specific sub-category and the number of genes in a sub-category. (**B**) COG classification. All 9,047 unigenes were aligned to 25 COG groups. The letters on the *x*-axis indicate the COG categories as listed on the right of the histogram, and the *y*-axis indicates the number of unigenes in category.
